# Neural Correlates of Face and Object Perception in an Awake Chimpanzee (*Pan Troglodytes*) Examined by Scalp-Surface Event-Related Potentials

**DOI:** 10.1371/journal.pone.0013366

**Published:** 2010-10-12

**Authors:** Hirokata Fukushima, Satoshi Hirata, Ari Ueno, Goh Matsuda, Kohki Fuwa, Keiko Sugama, Kiyo Kusunoki, Masahiro Hirai, Kazuo Hiraki, Masaki Tomonaga, Toshikazu Hasegawa

**Affiliations:** 1 Graduate School of Arts and Sciences, The University of Tokyo, Tokyo, Japan; 2 Japan Society for Promotion of Sciences, Tokyo, Japan; 3 Great Ape Research Institute of Hayashibara Biochemical Laboratories, Inc., Tamano, Japan; 4 Department of Human Relations Studies, School of Human Cultures, The University of Shiga Prefecture, Hikone, Japan; 5 Department of Psychology, Queen's University, Kingston, Ontario, Canada; 6 Section of Language and Intelligence, Primate Research Institute, Kyoto University, Inuyama, Japan; 7 Department of Psychology, Keio University, Tokyo, Japan; University of Leuven, Belgium

## Abstract

**Background:**

The neural system of our closest living relative, the chimpanzee, is a topic of increasing research interest. However, electrophysiological examinations of neural activity during visual processing in awake chimpanzees are currently lacking.

**Methodology/Principal Findings:**

In the present report, skin-surface event-related brain potentials (ERPs) were measured while a fully awake chimpanzee observed photographs of faces and objects in two experiments. In Experiment 1, human faces and stimuli composed of scrambled face images were displayed. In Experiment 2, three types of pictures (faces, flowers, and cars) were presented. The waveforms evoked by face stimuli were distinguished from other stimulus types, as reflected by an enhanced early positivity appearing before 200 ms post stimulus, and an enhanced late negativity after 200 ms, around posterior and occipito-temporal sites. Face-sensitive activity was clearly observed in both experiments. However, in contrast to the robustly observed face-evoked N170 component in humans, we found that faces did not elicit a peak in the latency range of 150–200 ms in either experiment.

**Conclusions/Significance:**

Although this pilot study examined a single subject and requires further examination, the observed scalp voltage patterns suggest that selective processing of faces in the chimpanzee brain can be detected by recording surface ERPs. In addition, this non-invasive method for examining an awake chimpanzee can be used to extend our knowledge of the characteristics of visual cognition in other primate species.

## Introduction

Examining the neural system of our closest living species, the chimpanzee, may provide valuable information towards understanding the proximate and ultimate causes (i.e. the physiological and evolutionary origins) of human cognition. Primates are considered to depend more on visual than olfactory cues for social information processing [1]. This paper reports the attempts of real-time measurement of neural activity during visual processing in an alert chimpanzee while she was observing meaningful stimuli.

Various aspects of chimpanzee visual cognition have been behaviorally explored, including categorical color perception [2], visual illusions [3], perception of object unity [4], global and local processing of visual elements [5], [6], and recognition of pictorial representations [7]. The recognition of faces is of particular importance for both chimpanzees and humans. A recent eye-tracking study revealed that chimpanzees view faces of animals in a human-like manner, gazing at the faces of conspecifics, humans, and other animals longer than environmental scenes and other parts of their bodies [8]. Other behavioral studies have revealed that the ability of chimpanzees to recognize faces is close to that of humans. Chimpanzees have sophisticated abilities for individual identification in terms of kinship detection [9] and understanding conspecific facial expression [10]. Moreover, in common with humans, they show disrupted processing for inverted faces [11].

Until recently, vast knowledge of the primate visual system has been primarily obtained from the brains of macaque monkeys and humans. Previous research has suggested that the ventro-lateral parts of higher visual areas, particularly the superior temporal sulcus and the inferior temporal cortex, are central for object and face recognition [12], [13], [14]. Electrophysiological studies in humans using surface-skin event-related potentials (ERPs) have shown a pattern of activity that is sensitive to object and face recognition at lateral occipital sites. This ‘N170’ component is a negative deflection that peaks between 140 and 200 ms after stimulus onset, which is generally larger in response to human faces compared to many other object categories [15], [16]. More recently, a magnetoencephalography study reported that face-selective neural activity appeared at a latency of 100 ms after stimulus onset [17]. These findings suggest that characteristic face processing activity in the human visual cortex begins after 100–200 ms following stimulus presentation. In the monkey brain, previous studies suggest that this processing begins less than 100 ms following stimulus presentation [18], [19], [20], although most studies have recorded activity at the cellular level rather than measuring ERPs. As for chimpanzees, the neural activity underlying visual processing has rarely been directly examined, until recently.

Several research groups have now conducted cognitive neuroscience studies in chimpanzees. These studies have demonstrated that the neural activity of chimpanzees is similar to that of humans during stimulus-independent resting states [21], [22], auditory perception [23], [24], communicative interaction [25], and visual processing [26], [27].

Parr and colleagues [27] recently conducted a functional neuroimaging study of chimpanzees to examine their neural activity for face recognition using positron emission tomography (PET). Five chimpanzees given an isotope marker performed a matching task with conspecific faces and objects. The subjects were then sedated before their brains were scanned. The results of the PET scan revealed that the superior temporal sulcus, the inferior temporal regions, and other vision-related areas showed significantly greater activity in the face-matching task compared to the object-matching task.

The results of Parr et al [27] uncovered the spatial location of brain activity in chimpanzee face recognition. However, measurement of neural metabolism with methods such as PET sheds little light on the temporal aspects of the neural processing of faces and objects in chimpanzees. In a pioneering neural examination of chimpanzee visual processing, Boysen and Berntson [26] measured ERPs from two sedated juvenile chimpanzees, using stereoscopic flashes as stimuli. Flash-evoked ERPs were recorded from two midline electrode sites, and the waveforms of the ERPs on the occipital midline were found to be comparable to those of humans. Unfortunately, the activity in the lateral-posterior cortical regions was not examined, although activity in these lateral regions is known to be essential for face and object recognition. Critically, ERP responses to images of meaningful objects, such as faces, have never been examined.

We have developed a protocol using non-invasive scalp ERP measurements in an adult female chimpanzee in her fully awake state, without a sedation procedure. We previously reported an examination of her ERPs in response to auditory stimulation [28], [29]. Our findings showed that the chimpanzee elicited a neural activity pattern indicating automatic detection of change in auditory stimuli, similar to a well-known pattern in humans known as ‘mismatch negativity’ [28]. Moreover, selective neural responses to the vocal sound of the subject's own name were observed, in comparison to vocalization of other individuals' names [29]. These findings demonstrate the usefulness of the sedation-free ERP measurement in investigating the neural basis of cognitive dynamics in the chimpanzee brain.

The current report describes examinations of visually evoked ERPs from the same chimpanzee, in response to observing objects and faces while in a fully awake state. In Experiment 1, photographs of human faces and spatially-randomized images generated from the same photographs were presented to the subject. In Experiment 2, pictures of objects from three categories (cars, flowers and faces) were presented. These experiments aimed to 1) measure the basic morphology of chimpanzee ERPs for face and object perception, 2) examine the time-course of electrophysiological activity that is specific to faces among the stimulus categories.

## Materials and Methods: Experiment 1

Experiment 1 was the first examination of visual ERPs elicited by an alert chimpanzee, while observing two classes of visual stimuli on a monitor. The stimuli consisted of three photographs of human faces and three photographs that were made by scrambling the three face photographs. The latter stimuli were matched to the face stimuli in luminance and color values. Since it is known that objects and faces are primarily processed in the posterior-temporal regions in the macaque and human brains [30], measurement electrodes were placed at occipito-temporal sites, as well as three midline sites.

### Chimpanzee subject

The subject was a female chimpanzee (*Pan troglodytes*) named Mizuki. She is currently housed at the Great Ape Research Institute of Hayashibara Biochemical Laboratories, Inc., Okayama, Japan, with other group members (two males, two females, and an infant). She was raised by human caregivers from a few days after birth. Since she arrived at the Great Ape Research Institute when she was 2 years and 1 month old, she has spent the majority of her time with other chimpanzees outside and inside compounds.

At the time of experimentation, Mizuki was 10 years old and had undergone other behavioral cognitive experiments [31], [32], as well as earlier ERP experiments [28], [29].

This research was conducted in accordance with the “Guide for the Care and Use of Laboratory Animals” of Hayashibara Biochemical Laboratories, Inc., and the Weatherall report, “The use of non-human primates in research”, since only non-invasive electroencephalography technique was utilized to measure the subject's neural activity. The research protocol was approved by the Animal Welfare and Animal Care Committee of The University of Tokyo and Hayashibara Biochemical Laboratories, Inc. (GARI-051101).

### Apparatus and stimuli

The experimental room consisted of concrete walls, with moderate lighting. The subject sat on a concrete platform and was fully awake during recordings. A 17-inch CRT display (IIyama LA702U) was set up in front of her approximately 40 cm away, at the horizontal level of her head. An infrared video camera was fixed on top of the CRT display to monitor the subject from a frontal view. We used this camera to check if the subject's gaze was directed to the stimulus display.

**Figure 1:**
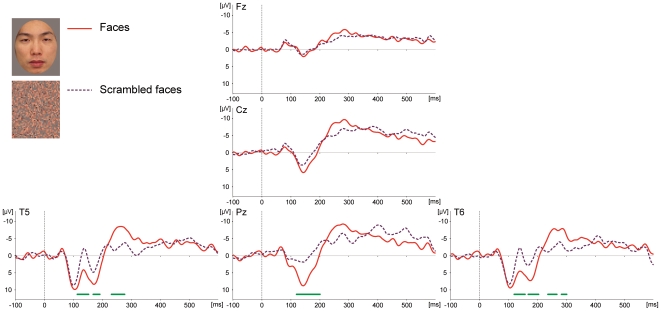
Averaged ERP waveforms elicited by faces and scrambled faces in Experiment 1. Numbers of trials in the analysis were 100 (faces) and 81 (scrambled faces). The light green bar indicates time points where waveforms differed significantly (over 20 consecutive time-points with p<0.05).

Experimental stimuli were color photographs of the faces of three Japanese adult males novel to Mizuki (see Figure 1 for examples of the stimuli). The images were digitally processed in 24-bit color by graphics software so that each face had the same oval shape, which were overlaid upon a gray rectangle (13° high and 11° wide). Three scrambled images used as control stimuli were created by dividing the face photographs into 50 fragments both in vertical and horizontal dimensions, and spatially scrambling the layouts of the mosaics (Figure 1), so that the luminance (146.65 cd/m^2^) and the number of pixels in the images (75000) were equal between stimulus categories. All the stimuli were displayed on a black background.

### Procedure

The experiment consisted of six blocks with 108 trials in each block. On each trial, one of the six stimuli was presented for 500 ms in a randomized order, and was followed by a 700 ms inter-stimulus interval consisting of an empty black screen. Between blocks, Mizuki was given a rest of ∼1 min, which allowed her to make considerable body-movements and to receive fruit rewards. During the recordings, an experimenter (one of Mizuki's caregivers) stood beside her to keep her still and facing the display. Occasionally Mizuki's gaze appeared to avert from the monitor. When this occurred, another experimenter, who was monitoring the subject's gaze direction, manually added a marker in the EEG data via a keyboard connected to the measurement computer.

### ERP recording and analysis

EEG was recorded from Ag/AgCl electrodes attached to five scalp positions (Fz, Cz, Pz, T5, and T6), according to the international 10–20 system for humans. The signals were referenced to the forehead midline (FPz). Visual ERP recordings in humans often use a reference electrode on the subject's nose, as it is far from the sites of interests (visual areas around posterior brain regions). However, this study used a reference electrode on the Fpz, so that the subject was not distracted by the lead and electrode on her nose. A ground electrode was positioned at the left earlobe.

The electrodes were filled with Quick GEL and impedances were kept below 6 kΩ. Signals were amplified by NuAmp-40 and processed by Acquire 4.3 software (NeuroScan Inc.) with a 1,000 Hz sampling rate. A 0.1–30 Hz band-pass filter (24 dB/oct) was applied in the offline analysis. All data were segmented into 700-ms epochs, including a 100-ms pre-stimulus baseline period, based on time markers of the stimulus onset. These epochs were baseline-corrected with respect to the mean amplitude over the 100-ms pre-stimulus period. Epochs that exceeded ±60 µV were excluded from analysis. Epochs that contained ‘non-looking’ markers described above were also excluded in the analysis. The numbers of epochs accepted for the analysis were 100 for the faces and 81 for the scrambled faces.

A successive analysis of variance (ANOVA), with a single factor of stimulus type was applied along each data point of each channel to test differentiation among the potentials for stimulus categories. To avoid the detection of spurious differentiation between categories, we considered a time range of 20 consecutive time points (20 ms) of p-values <0.05 to indicate a significant difference.

## Results and Discussion: Experiment 1

### ERP Morphology

ERP waveforms elicited by both faces and scrambled faces showed similar morphologies, as depicted in Figure 1. These waveforms can be grouped into two electrode sites: midline and lateral regions. Among the midline sites (Fz, Cz, and Pz), the waveforms showed three dominant deflections. Early negative deflections peaking at around 80 ms post-stimulus (averaged latencies across the stimulus types were 81 ms at Fz, 80 ms at Cz, and 71 ms at Pz) were followed by positive deflections peaking at around 140 ms (142 ms at Fz, 141 ms at Cz, and 138 ms at Pz). These deflections were followed by a late negative slow wave, which was visible after approximately 250 ms. At the lateral occipito-temporal sites (T5 and T6), the initial negative peaks (59 ms at T5, and 57 ms at T6) were followed by complex deflections, consisting of positive-negative-positive potentials (the latencies of each peak/valley at T5 (T6) sites were 107 (104) ms for the positive deflection, 137 (140) ms for the negative deflection, and 171 (173) ms for the second positive deflection). These deflections were followed by late negative slow waves, as were the cases at the midline electrodes.

### Effects of stimuli

Significant differentiations on the ERPs between stimulus types were detected over the posterior electrode sites at Pz, T5 and T6, as indicated in Figure 1 (all F-values >3.89 [df1 = 1, df2 = 179], p-values <0.05). This differentiation was found in two time ranges. The first period ranged from 115 to 201 ms post stimulus, in which ERPs showed enhanced positive potentials for the face stimuli compared to the scrambled stimuli. The second period ranged from 234 to 297 ms post stimulus, where the face-evoked negative deflection was more prominent than that elicited by scrambled faces. This modulation was observed around 237–274 ms post stimulus at T5, and 239–364 ms after stimulus onset at T6. Detailed latencies of these differentiations at each electrode position are depicted in Table 1.

**Table 1:** Time ranges where ERPs for faces and scrambled faces differed significantly in Experiment 1 (post stimulus latencies in msec).

Electrode sites	Pz		T5		T6	
Early period	119–200		115–153, 170–192		117–154, 164–201	
Late period			233–278		233–264, 280–297	

### Discussion of Experiment 1

Experiment 1 revealed the morphology of the subject's visually evoked ERPs among medial and lateral sites. The fundamental pattern shared by all electrode sites consisted of three periods: an early negative component (∼60–90 ms), a period of positive deflection (∼100–200 ms), followed by a negative deflection (after 200 ms). Within these patterns, the mid-latency period (100–200 time range) showed a different pattern between the midline and occipito-temporal regions. In this period, the waves from three midline electrode sites exhibited a large positive valley, while the occipito-temporal electrodes detected a ‘W-shape’ pattern with a central negative component occurring at approximately 140 ms.

The most substantial difference we found between the two stimulus types was a positive deflection around the posterior and lateral regions (Pz, T5, and T6). This effect was observed at approximately 120–200 ms. Another feature distinguishing the stimulus targets was restricted to the lateral occipital sites (T5 and T6), occurring after 237 ms post stimulus. ERP modulations were found to be more sensitive to stimulus type over posterior sites compared to anterior sites, and around occipito-temporal sites compared to midline sites.

It is possible that these ERP differentiations reflect face-specific neural processing in the chimpanzee brain. However, because the face stimuli contain rigid parts and configurations, whereas the compared stimuli (scrambled faces) did not involve these structural characteristics, it is possible that these divergences reflect differences in the processing of general objects, and are not specific to faces. The next experiment therefore examined the properties of the neural differentiation between stimulus types in Experiment 1 in more detail.

## Materials and Methods: Experiment 2

Experiment 2 had two major aims. First, we sought to replicate the basic morphology of ERPs elicited in Experiment 1. Second, we hoped to further examine possible face-specific activity in the chimpanzee's visual ERPs. In this experiment, ERPs elicited by face stimuli were compared with those in response to other objects (flowers and cars). These stimuli contain lines and subparts, similar to those in face stimuli, and have been previously utilized in experiments of face and object processing in chimpanzees and monkeys [33], [34].

All of the methodology in Experiment 2 was identical to Experiment 1, with the exception of the differences in stimuli described below.

### Stimuli

**Figure 2:**
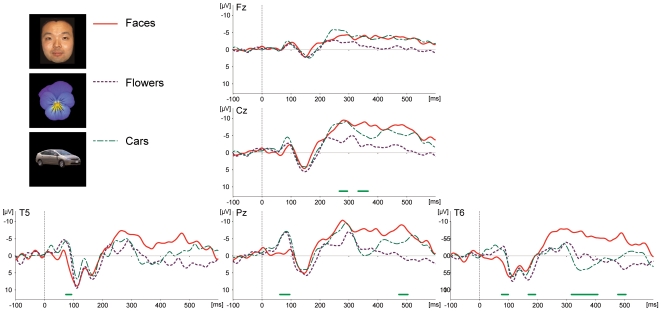
Averaged ERP waveforms elicited by faces, flowers, and cars in Experiment 2. Numbers of epochs for the analysis were 69 (faces), 64 (flowers), and 71 (cars).

Three photographs for each of three categories (faces, flowers, and cars) were used as stimuli (see Figure 2 for examples of the stimuli). The face images were photos of different Japanese adult males to those in Experiment 1, and were novel to Mizuki. Viewpoint and size of objects were virtually matched within each category, because it has been suggested that the human ERP component to faces (i.e. N170) is influenced by interstimulus perceptual variance, and that this factor should be controlled to examine face-sensitivity in the neural activity [35]. The averaged luminance (mean, 120.35 cd/m^2^; SD, 0.39 cd/m^2^) and pixel size (mean, 89670.11, SD; 1019.46) among categories were matched, varying less than 0.7%. Each photograph from each of 3 types ×3 categories was presented 72 times in a randomized order. The experiment consisted of 648 trials divided into six blocks. The numbers of epochs accepted for the analysis were 69, 64, and 71 for the face, flower, and car stimuli, respectively.

## Results and Discussion: Experiment 2

### ERP morphology

Figure 2 illustrated the ERP waveforms elicited by the three stimulus categories. For all categories of stimuli, the ERP morphology was similar to that elicited in Experiment 1. Waveforms from the midline sites initially exhibited negative variations peaking around 90–100 ms (averaged latencies across the stimulus types were 98 ms at Fz, 95 ms at Cz, and 94 ms at Pz), followed by positive variations peaking around 150–160 ms (157 ms at Fz, 154 ms at Cz, and 154 ms at Pz), and subsequent sustained negative variations which were apparent after approximately 250 ms post stimulus. For the lateral occipito-temporal sites (T5 and T6), the early negative components peaking at 78 ms at T5 and 75 ms at T6, exhibited W-shaped deflections, where the latency of each peak/valley at T5 (T6) sites was 113 (110) ms for the first positive deflection, 138 (138) ms for the negative deflection, and 162 (162) ms for the second positive deflection. These patterns were followed by late negative slow waves, as were the cases at the midline regions.

### Effects of stimuli

ERPs were found to be sensitive to stimulus category in several time ranges (all F-values >3.081 [df1 = 2, df2 = 201], p-values <0.05). The detailed time ranges of the significant main effects of stimulus type are presented in Table 2. A main effect of stimulus appeared at a period of as early as 62–98 ms at Pz and T6 sites. This early period of differentiation reflected the faces eliciting positive potentials relative to the other categories at posterior and temporal sites. *Post-hoc* direct comparisons of faces vs. flowers, and faces vs. cars also confirmed that the potentials in this period were significantly different (all F-values >3.91, p-values <0.05). In a middle time range of 168–192 ms, there was a main effect of stimulus at the T6 site, but this period did not display face-specific modulation. In the following period after 270 ms, where negative slow waves were prevalent across the recording sites, enhanced negative potentials were distributed for faces relative to the other categories at the Cz, Pz, and T6 sites. The *post-hoc* tests revealed that some periods (325–362 ms and 476–498 ms) at T6 were indeed differentiated significantly in both comparisons of faces vs. flowers and faces vs. cars.

**Table 2:** Time ranges where ERPs showed a main effect of stimulus type in Experiment 2 (post stimulus latencies in msec).

Electrode sites	Cz		Pz		T6	
Early period			62–95		76–98, 168–192	
Late period	270–297, 335–368		472–503		316–407, 476–504	

### Discussion of Experiment 2

The ERPs observed in Experiment 2 exhibited waveforms similar to those in Experiment 1. The potential deflections consisted of three periods: an early negative component (in a period within 100 ms post stimulus), a period of positive deflection (in a period ∼100–200 ms), and a subsequent negative deflection (after 200 ms latency). The waveforms at the occipito-temporal electrodes exhibited a W-shaped pattern in the mid-latency period (100–200 ms time range). Significant differences between stimulus categories were detected at sites other than the frontal midline. Among these differences, the early period within 100 ms and the later period after 270 ms post stimulus showed significant face-specific differences. The face stimuli elicited an enhanced positivity in the early period within 100 ms latency, and an enhancement of the later negative potential around the posterior and lateral sites. All of these results are similar to those observed in Experiment 1.

However, some differences were apparent between Experiments 1 and 2. First, the time course of the differentiation between stimulus categories was not aligned between the experiments. The onset latency of the waveforms' divergences in Experiment 2 (62ms) was shortened in comparison to Experiment 1 (115 ms). On the other hand, the period of later differentiation revealed that Experiment 2 was delayed compared to the first experiment. This misalignment of latencies might be due to differences in the categories of non-face stimuli between the two experiments, although this issue needs further clarification.

Overall, Experiment 2 replicated the morphological pattern of visually evoked ERPs and the existence of face-specific activity observed in Experiment 1, although the detail of these face-specific electrophysiological differences requires further investigation.

## Discussion: General

This study examined visually evoked ERPs in an awake chimpanzee while observing faces and objects. The results showed that there were common ERP patterns in response to several types of stimuli, confirming that the subject successfully perceived the stimuli throughout the experiments. Furthermore, the elicited ERPs indicated face-sensitive activity in posterior and lateral recording sites.

The ERP waveforms elicited in the two experiments exhibited a relatively similar morphology in response to all stimuli. The previous study examining chimpanzees' visual ERPs, Boysen & Berntson [26] recorded flash-evoked ERPs from sedated subjects. It was found that successive components at a Cz site exhibited latencies of approximately 70, 125, and 225 ms post stimulus with positive – negative – positive polarities respectively. In the present data, waveforms elicited at the midline electrodes showed components with peaks at approximately 90, 150, and 250 ms, with negative – positive – negative polarities respectively. This previous study, however, utilized different reference channels than our present experiments (the left earlobe was used in the previous study, whereas the forehead was used in the current study), which may be responsible for the difference in polarities of the deflections. In addition, the displacement in latency may be due to differences in stimuli between studies (flashed lights in the former study and meaningful objects in the present study), although the time-course of the ERP pattern requires further elucidation with a larger sample. In addition to these results from midline sites, our data also examined the occipito-temporal ERP pattern elicited by faces and object stimuli.

The characteristic morphology of ERPs from occipito-lateral sites was observed at a post stimulus latency of approximately 100–200 ms. In this time range, the waveforms obtained from the three midline electrode sites displayed a large positive valley, while the occipito-temporal electrodes exhibited a W-shaped pattern with a central negative component at approximately 140 ms latency. Although the measurement of ERPs generally provides little information about the precise spatial locations of the neural origin of measured signals, it seems reasonable that the dissociation in the ERP morphologies between medial and lateral regions might reflect the dorsal and ventral dissociation in visual processing in the primate cortex [30]. Boysen & Berntson [26] suggest that the waveforms of the chimpanzee visual ERP have some broad similarities with those of humans. Typical human ERPs in visual perception have several components (e.g. P1, N1, P2, and N2) within ∼250 ms latency range. These are followed by one or two slow and large potentials (e.g. the P300). The chimpanzee data in the current study (as well as the results of Boysen & Berntson) show a similar pattern, since deflections were observed within a 250–300 ms latency, preceding the range of a later large potential shift.

The current experiments showed that ERPs evoked by face stimuli significantly differentiated from those elicited by other stimulus categories. The differences between stimulus types were observed in posterior regions. In the early time range within 200 ms post stimulus, face-evoked ERPs showed enhanced positivity, while in the time range of later negative variations, face ERPs showed enhanced negativity.

The advantage of electrophysiological methods is the high temporal resolution they offer in the examination of neural activity. Previous human ERP research has demonstrated that the N170 component can be used as an index of face-specific neural processing [15], [16]. The latency of the N170 component suggests that it begins at around 140–200 ms in the human cortex [15]. A recent magnetoencephalography study reported that face-selective neural activity appears at a latency of 100 ms following stimulus onset [17]. In the current experiments, the earliest onset of ERP differentiation was 115 ms in Experiment 1, and 62 ms in Experiment 2. These findings suggest that the neural classification between faces and other categories occurs with an overlapping time range between chimpanzees and humans, although there is an indication that this differentiation might be earlier in the chimpanzee relative to the human brain.

Comparing chimpanzee ERPs elicited by faces with the findings of human studies raises the question of whether any chimpanzee homolog of the human N170 potential exists. In the current study, the lateral occipital sites (T5/T6) revealed a negative component peaking at ∼140 ms post stimulus. The time range of this component was significantly different between stimulus types in Experiment 1, but did not display the enhanced negativity in responding to the faces typical of the human N170. In Experiment 2, this time range did not even show clear specificity to stimulus type. Taking these results into account, we conclude that the present study did not reveal a chimpanzee homolog of the human N170.

Previously, we reported that a chimpanzee subject generated an ERP that we considered homologous to the human mismatch negativity potential [28]. Mismatch negativity has previously been reported in other animals such as monkeys [36] and mice [37]. In contrast, to our knowledge, N170-like ERPs have never been reported among animals other than humans. Pineda et al. [20] reported visual ERPs that were elicited by monkeys observing face stimuli. They found that electrodes at lateral-temporal sites (T3/T4) showed large negative components that peaked around 100 ms post stimulus, and were more prominent in response to monkey compared to human faces. Although their study did not examine the occipito-temporal sites, the reported ERPs (at the midline and lateral temporal regions) appeared to have no N170-like component after 100 ms latency.

It should be noted that surface ERP patterns are susceptible to many physiological factors, such as speed of signal propagation between the neurons and structures of the cortex. It is also known that the N170 is not observed in human infants at the occipito-temporal regions; instead, some late components (N290 and P400) are considered to reflect face-selective neural responses in infants [38], [39], [40]. This difference in ERP response may be due to a difference in the speed of signal transfer along axons because myelination is still developing in infancy [41]. In comparing humans with chimpanzees, structural difference is another factor that may have a substantial influence, in addition to the issue of the processing speed. While the chimpanzee brain has a structure comparable to that of humans, the shapes of the bone and muscles of the skull are substantially different to humans' [42]. Although a mismatch negativity was observed in the midline regions of the chimpanzee [28], this does not ensure that the other activity involved in generating human ERPs was also present in the chimpanzee ERPs. Thus, the apparent lack of an N170 in this study does not necessarily indicate major differences in the processing of faces between chimpanzees and humans.

Many human studies have reported that faces involve more right- than left-hemisphere processing [15], [43], [44], but such lateralization has not been clearly shown in the monkey brain [45]. In the current study, Experiment 2 displayed face-specific activity that was right-lateralized, reflected in the enhanced late negativity occurring only at the T6 site. However, Experiment 1 did not show any significant right-dominant face-sensitive activity. Thus, we cannot currently draw firm conclusions from these findings about the existence of lateralization for face processing. It should be noted that a recent PET study by Parr et al. [27] suggested that cortical regions in the chimpanzee showed face-specific metabolic changes with right-hemispheric dominance. The data of Parr et al. [27] and our study suggest hemispheric lateralization of visual processing in the chimpanzee brain exists, but is weaker than that in the human brain. However, this conclusion requires further validation.

Finally, several practical and interpretational limitations of this study should be noted. First, because the subject's gaze was manually monitored during the recording, small eye movements of subject could not be controlled in the analysis. This could potentially reduce the clarity of the ERP waveforms. A more sophisticated non-invasive eye tracking system should be employed in the future examinations of primate visual ERPs to remove this potential confound.

Second, the data could be influenced by the familiarity of stimulus categories, in accord with findings that primate visual areas are sensitive to stimulus familiarity [46], [47], [48]. The present subject, Mizuki, was a captive animal who interacted with human staff at the research institute every day. The subject had also been involved in a previous behavioral experiment involving face recognition (Hirata, unpublished data). At the same time, it should be noted that the non-face objects used in this study (flowers and cars) were not unfamiliar categories for the subject. The subject's living environment at the research institute allowed her to have experience observing objects within all visual categories used in this study. Still, the degree of familiarity of the stimulus categories could be better controlled.

Third, this study used only human faces as stimuli, meaning that ERPs for perceiving chimpanzee faces were not examined. Whether or not primates have a specialized mechanism for perceiving the faces of conspecifics has been the subject of much debate [49], [50]. The present results did not reveal an N170-like component in the chimpanzee, but it is possible that such a component would appear if the subject was presented with conspecific faces instead of human faces. Previous studies have produced inconsistent results regarding the question of whether primates differentially process conspecific and non-conspecific faces. Some findings have suggested a difference [51], [52], [53], but some research has suggested that nonhuman primates process human faces in a similar manner to conspecific faces [8], [11], [54]. This controversy is likely to be related to environmental factors, such as the effects of expertise [49], [52]. Our next study will examine the issues mentioned here, by exploring ERPs elicited by the faces of familiar and non-familiar conspecifics, to further clarify face-sensitive neural activity in the chimpanzee.

## Conclusion

The present study reported ERP patterns associated with visual object and face perception in a fully awake chimpanzee. ERPs evoked by face stimuli were differentiated from those of other categories, and our results indicate that this differentiation occurred in the same time range, or possibly even earlier, than in humans. This activity was observed as an early (within 200 ms) enhanced positivity and a late (after 200 ms) enhanced negativity at posterior and lateral regions. The exact time ranges and location of this face-specific ERP component remain to be examined in further detail. Together with recent studies of chimpanzee functional brain activity [22], [24], [27], [28], [29], our non-invasive physiological examination will aid further investigations into the proximate and ultimate causes of human cognition.
